# Supplementary material for: Crystallographic and Physicochemical Analysis of Bovine and Human Teeth Using X-ray Diffraction and Solid-State Nuclear Magnetic Resonance
Source: J Funct Biomater. 2022 Nov 19;13(4):254. doi: 10.3390/jfb13040254 (PMC9680385; doi:10.3390/jfb13040254)
Supplement: Supplementary file 1 [file jfb-13-00254-s001.zip › Table S1.pdf]

Table S1. The hexagonal symmetry-P63/m space group. Retrieved from Journal of Applied Crystallography, 43,320-327,2010.

**a**	**b**	**c**	**α**	**β**	**γ**
9.421	9.421	6.88	90	90	120

**Atom**	**Site**	**Occ**	**x**	**y**	**z**	**U**
Ca	4f	0.33333	0.33333	0.6667	0.00092	0.019
Ca	6h	1	0.24525	0.99333	0.25	0.019
P	6h	1	0.39811	0.36952	0.25	0.019
O	6h	1	0.32711	0.48474	0.25	0.019
O	6h	1	0.58616	0.465	0.25	0.019
O	12i	1	0.43152	0.25583	0.07261	0.019
O	4e	0.5	0	0	0.18819	0.019
