# Supplementary figures and images for: Crystallographic and Physicochemical Analysis of Bovine and Human Teeth Using X-ray Diffraction and Solid-State Nuclear Magnetic Resonance
Source: J Funct Biomater. 2022 Nov 19;13(4):254. doi: 10.3390/jfb13040254 (PMC9680385; doi:10.3390/jfb13040254)

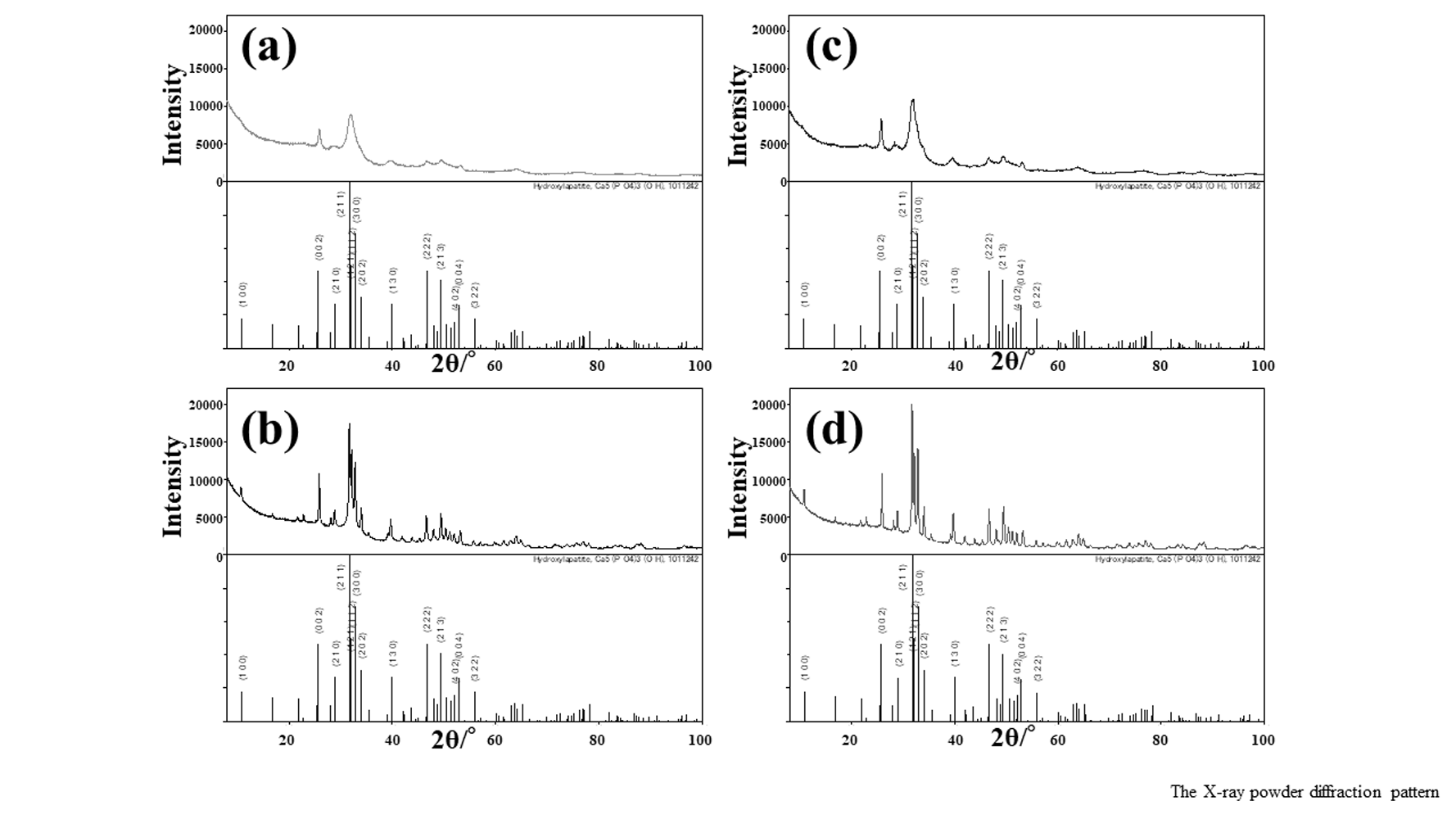

Supplement: Supplementary file 1 [file jfb-13-00254-s001.zip › Figure S1.tif]

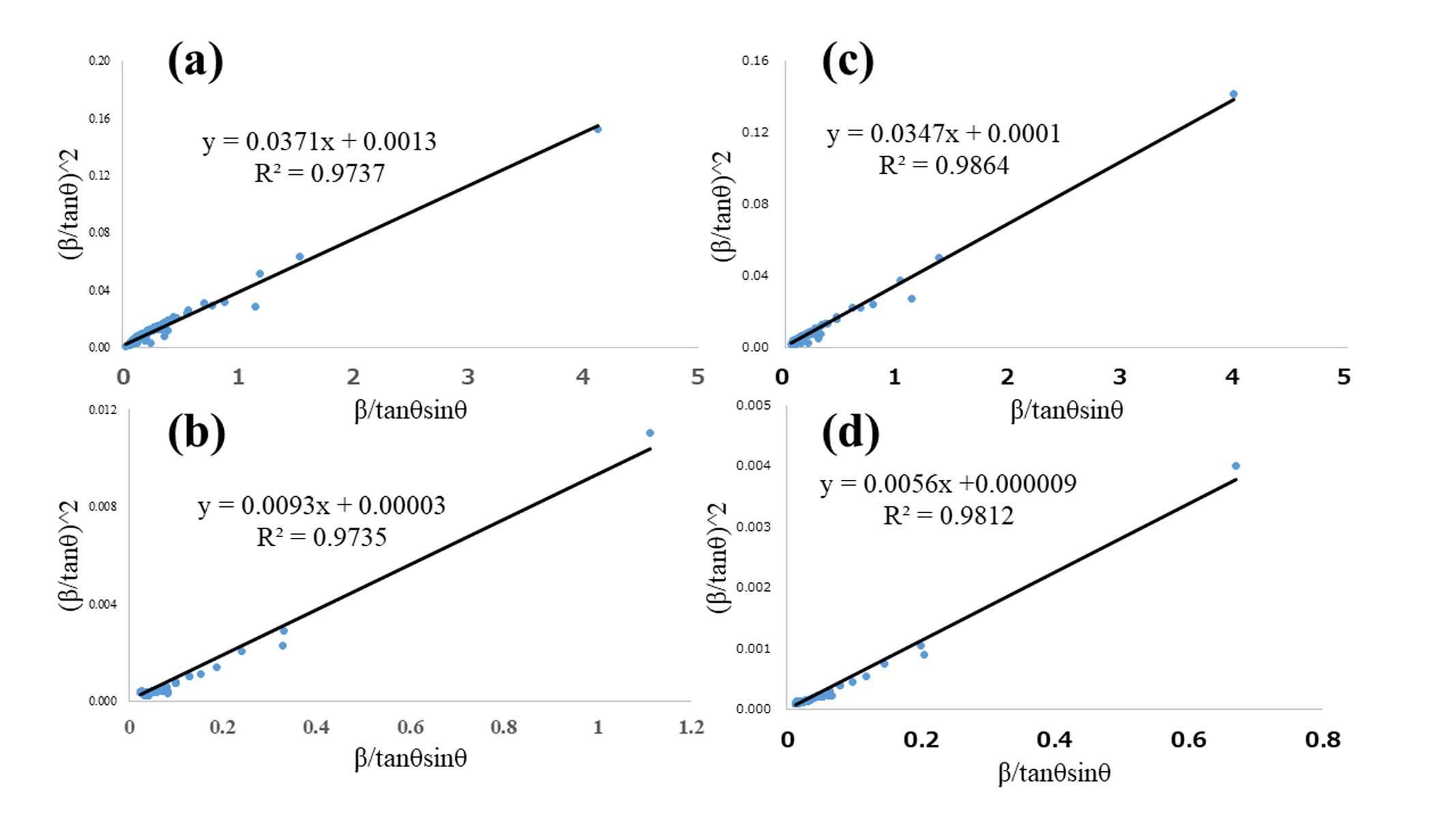

Supplement: Supplementary file 1 [file jfb-13-00254-s001.zip › Figure S2.tif]
